# Waterbird zoogeochemical effects shape wetland nutrient dynamics and algal communities in Laurentian Great Lakes coastal freshwater wetlands

**DOI:** 10.1111/1365-2656.70308

**Published:** 2026-07-08

**Authors:** Taylor C. Michael, Paisley Kostick, Matthew A. Wuensch, David M. Costello

**Affiliations:** ^1^ Department of Biological Sciences Kent State University Kent Ohio USA; ^2^ Department of Biological Sciences University of Alabama Tuscaloosa Alabama USA; ^3^ Department of Biology University of North Dakota Grand Forks North Dakota USA

**Keywords:** biogeochemistry, consumer‐derived nutrients, excretion, nitrogen, phosphorus, phytoplankton, waterfowl

## Abstract

1. Freshwater wetlands along the Laurentian Great Lakes provide vital ecosystem services, two of which include providing habitat for millions of waterfowl and reducing nutrient (nitrogen (N) and phosphorus (P)) runoff from the landscape. Nutrient bioavailability in wetlands is influenced by waterbirds, which shape biogeochemical dynamics through the excretion of N‐ and P‐rich guano.
2. There has been limited focus on how waterbirds shape nutrient budgets (i.e. via zoogeochemical effects) in wetlands that are managed for both waterfowl habitat and nutrient retention. Therefore, we examined how three distinct functional groups of common waterbirds influence nutrient biogeochemistry and algal community structure in two coastal freshwater wetlands along Lake Erie (Ohio, USA).
3. We experimentally measured how waterbird‐derived nutrient contributions shape wetland nutrient removal and elicit changes in algal community composition through experimental additions of guano to microcosms. We collected biweekly waterbird densities throughout 1 year and used guano nutrient content and dropping rates to estimate annual waterbird N and P fluxes. We then assessed the relative importance of waterbird‐nutrient contributions through comparisons to previously monitored net nutrient loads from landscape runoff.
4. Waterbird guano additions initially increased nutrient concentrations in microcosms; however, wetland sediments rapidly removed most of the total P, but only approximately half of the total N from the water column. Waterbird guano from different functional groups favoured distinct algal communities, driven primarily by differences in guano total N content. Waterbird‐derived nutrient fluxes were highest during fall migration and may be locally important in wetlands during nutrient‐limiting times. However, annual waterbird‐derived nutrient fluxes are often negligible (<1.3%) compared with external loads from surrounding agriculture‐dominated watersheds.
5. By understanding the zoogeochemical effects that waterbirds have in freshwaters, we can inform management decisions that simultaneously protect waterfowl habitat and enhance nutrient removal processes to maximize multiple ecosystem services in the Great Lakes region.

## Graphical abstract

Distinct waterbird species influence nutrient cycling through guano contributions in Great Lakes coastal wetlands. Waterbird‐derived nutrients favour distinct algal communities and wetland sediments rapidly remove waterbird nutrients via biogeochemical processes. These contributions may be locally important during nutrient‐limiting times, but the magnitude of nutrients contributed depends on wetland management strategy.

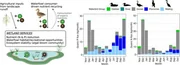

## 1 INTRODUCTION

Wetlands provide essential breeding grounds and stopover points for numerous waterfowl species, many of which are currently experiencing population declines globally (U.S. Fish and Wildlife Service, 2022; Johnson & Owen, 1992; Zimpfer et al., 2012). It is common for migratory populations of waterfowl to stopover and congregate in wetlands along their routes, which offer an abundance of shelter and food (O'Neal et al., 2012). Waterfowl and other waterbirds translocate large subsidies of nutrients (i.e. nitrogen [N] and phosphorus [P]) between ecosystem boundaries (Post et al., 1998) and alter the form of nutrients (organic vs. bioavailable) through feeding and excretion (Kitchell et al., 1999; Pettigrew et al., 1997; Vizzini et al., 2016). Nutrients can also be ingested and released within a wetland via consumer‐driven nutrient recycling (Atkinson et al., 2017; Vanni, 2002), influencing the bioavailability of nutrients for primary producers. Therefore, waterbirds can play a large role in transporting nutrients to and from wetlands and altering the bioavailability of nutrients within wetlands (Kitchell et al., 1999; Pettigrew et al., 1997). Through zoogeochemical effects, animals influence biogeochemical processes and alter ecosystem‐level nutrient cycles (Lopez et al., 2026; Schmitz et al., 2018). Nutrient inputs from animals, such as waterbirds, can have profound impacts on the ecological structure and function of aquatic ecosystems, including the alteration of nutrient cycling processes, nutrient export and primary production rates, with cascading effects on habitat quality for all organisms.

Waterbird guano is rich in N and P and can stimulate algal and aquatic vegetation growth and stimulate bottom‐up effects on food webs (Petkuviene et al., 2019; Pettigrew et al., 1997; Vizzini et al., 2016). Furthermore, the relative supply of N and P in surface waters can shape algal community composition (e.g. green algae and cyanobacteria) (Tilman et al., 1982). Waterbirds of different functional guilds (e.g. diet and biomass) can have distinct nutrient contents in their guano. Guano nutrient stoichiometry vary among waterbird species but are typically relatively low in P in herbivorous species (Adhurya et al., 2020). Low P droppings may promote P limitation (high N:P) in surface water, which can limit phytoplankton biomass and favour diverse assemblages of algae, with the best competitors for P having the highest biomass (Tilman et al., 1982). Conversely, piscivorous species have high P guano which enhances N limitation, and may favour N‐fixing cyanobacteria (Jankowiak et al., 2019). Therefore, both guano inputs and ecosystem context are important in predicting zoogeochemical effects (Lopez et al., 2026; Subalusky & Post, 2019).

In nutrient‐limited systems, nutrient‐provisioning from waterbird guano can support food webs (Kitchell et al., 1999; Pettigrew et al., 1997). However, in nutrient‐rich systems, such as shallow areas of the Laurentian Great Lakes in North America, excess nutrient inputs can be detrimental and may contribute to eutrophic conditions that result in harmful algal blooms and hypoxic events (Jankowiak et al., 2019; Watson et al., 2016). In addition to providing waterbird habitat, Great Lakes coastal wetlands also provide critical nutrient removal services that lessen nutrient loads from the landscape before entering downstream waters (Herdendorf et al., 2006). Wetlands are hotspots of biogeochemical cycling and microbial nutrient removal processes like denitrification (N removal via nitrate reduction to nitrogen gas) and abiotic processes like long‐term phosphorus burial (Myers‐Pigg et al., 2023; Zedler & Kercher, 2005). Two primary goals of wetland management and restoration are either to provide waterfowl and marsh bird habitat or enhance nutrient removal and retention, but not often both in conjunction (Hansson et al., 2005). A better understanding of zoogeochemical impacts from waterfowl on ecosystem‐scale nutrient cycles is necessary to inform ways in which these management goals can simultaneously be met.

Within wetlands and other freshwater ecosystems, animals can play a large role in altering nutrient recycling and availability (Atkinson et al., 2017; Kitchell et al., 1999; Michael et al., 2023; Post et al., 1998; Stears et al., 2018; Vanni, 2002), although these contributions are infrequently considered in nutrient budgets and management decisions (Buchkowski, 2025; Michael et al., 2025). Limited research has examined waterbird zoogeochemical effects in wetlands (Lopez et al., 2026), with much of the existing work investigating arid regions with sparse, spatially isolated wetlands and high waterbird densities, that results in high waterbird‐nutrient loading (accounting for 40% of N inputs and 75% of P inputs; Andersen et al., 2003; Post et al., 1998). However, many other areas in North America have lower waterfowl densities and more abundant and dispersed wetland and lake area for waterfowl to use. There have been limited studies considering how waterbirds contribute nutrients to wetlands in the Great Lakes region, where there is moderately high wetland area, which facilitates short travel distances between distinct wetlands across the landscape, and results in moderate waterbird densities dispersed among wetlands. Additionally, this region contains high agricultural land use and thus experiences high nutrient runoff. While waterbirds contribute nutrients via excretion (Adhurya et al., 2020; Post et al., 1998), their contribution to freshwater nutrient budgets is not well understood. Direct study of wetlands in agriculture‐dominated landscapes is needed to understand the role of waterbirds on nutrient budgets and ecosystem function in ecosystems like Great Lakes coastal wetlands within the Mississippi flyway (Figure 1).

**FIGURE 1:**
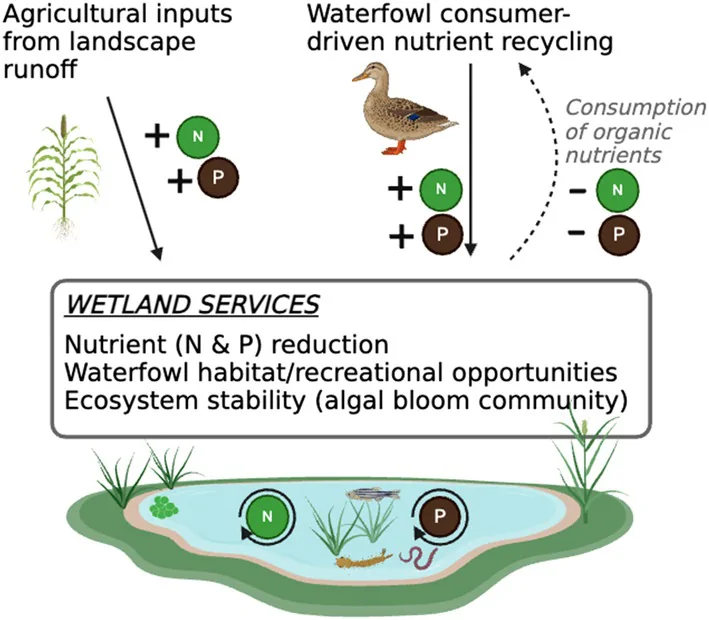
Conceptual diagram showing how nutrients from watershed inputs and waterbird excretion affect wetland ecosystem services.

We investigated how waterfowl shape nutrient recycling, algal communities, and wetland nutrient budgets through their guano contributions in coastal wetlands of Lake Erie. To address our questions we performed experiments using guano from three common waterbirds species in the region (i.e. mallard, Canada goose and double‐crested cormorant) that represent different functional groups, including body size (e.g. ranging from medium‐bodied cormorants and mallards to large‐bodied Canada goose), diet (e.g. omnivorous mallards, herbivorous geese and piscivorous cormorants; Boros, 2021), and excretion stoichiometry (e.g. low N:P cormorant guano vs. high N:P mallard and goose guano). Specifically, we had the following questions: (1) How effectively do wetland sediments remove waterbird guano‐derived nutrients from the water column and shape nutrient availability? (2) How does waterbird guano shape the phytoplankton community? and (3) What is the nutrient flux from waterbirds in wetlands and how do these waterbird‐derived fluxes compare to watershed runoff nutrient loads in two wetlands with contrasting management goals (i.e. nutrient removal vs. waterbird habitat)? We expected that wetland sediments would buffer most of the added nutrients from waterbirds through P retention and N removal processes, with differences among waterbirds driven by guano nutrient content (Adhurya et al., 2020; Vanni, 2002). We also expected that differences in guano N:P, driven by differences in difference in waterbirds functional traits, would cause variation in the phytoplankton communities. We expected that waterbirds would act as a source of nutrients to Great Lake's coastal wetlands, but that waterbird contributions would be small in comparison with nutrient loads from landscape runoff in agriculture‐dominated watersheds. Finally, we expected that the wetland managed for waterfowl habitat would have greater waterbird densities, and thus greater waterbird‐derived nutrient contributions, which would make up a small but relevant fraction of the nutrient load to the wetland and export to the lake. In contrast, we expected the wetland managed for nutrient retention would have lower waterfowl densities and nutrient contributions, that would be a smaller part of the total nutrient load and export.

## 2 MATERIALS AND METHODS

### 2.1 Study site

To address our questions, we conducted our research in two wetlands in northwest Ohio: Old Woman Creek National Estuarine Research Reserve (OWC) and Turtle Creek Bay Wetland (Turtle Creek) (Figure S1). OWC is a protected freshwater estuary with a large wetland complex (0.422 km^2^) that has an extensive watershed monitoring programme with a high‐resolution inflow and outflow nutrient dataset. Turtle Creek is a diked, water‐level managed wetland (0.635 km^2^) located within Magee Marsh Wildlife Area that is specifically managed for waterfowl and marsh bird habitat, which is common of many marshes throughout northwest Ohio. These wetlands have watersheds that are primarily composed of agricultural land use (62% at OWC, 77% at Turtle Creek; Herdendorf et al., 2006; Ottawa Soil and Water Conservation District, 2018), are connected to Lake Erie, close in proximity to other wetlands, and sit within the Mississippi flyway. By investigating these two wetlands, we can provide insight into the role of nutrient fluxes from waterbird guano in wetlands with primary management goals of either nutrient retention (OWC) or waterbird habitat (Turtle Creek).

### 2.2 Waterbird guano collection and nutrient analysis

We collected guano from waterbirds at the nearby Winous Point Marsh and OWC (Figure S1), where managing agencies have existing population monitoring efforts in place. All guano collections involving the handling of animals were completed under Winous Point Marsh Conservancy ‐ Permit #24398. We collected guano from three species common in Great Lake coastal wetlands: mallard (*Anas platyrhynchos*), Canada goose (*Branta canadensis*), and double‐crested cormorant (*Nannopterum auritum*). We collected wet mallard and Canada goose guano from trapped individuals. There were no ongoing trapping efforts of double‐crested cormorants by managing agencies that we collaborated with; therefore, we collected dry double‐crested cormorant guano (<1 day old) from beneath roosting locations. Dry cormorant guano may have lower N content (via possible microbial uptake and ammonium volatilization) and relatively less N as soluble forms (which could slightly delay nutrient release) relative to wet cormorant guano. Therefore, our results present a conservative estimate of cormorant effects. For each species, guano from multiple individuals was combined into composite samples to homogenize material and minimize variation in nutrient content of guano used in our experimental additions. For each guano type, we measured total phosphorus (TP), total nitrogen (TN), and total carbon (TC) content. To measure TP, we used acid digestion (5 mL of nitric acid and 2 mL of sulphuric acid to 0.4–0.6 grams of oven‐dried guano) at reflux temperatures (95°C) using a HotBlock until the volume reached ~1 mL, and then diluted and analysed samples via ICP‐OES. TN and TC were measured using an elemental analyser (Costech Analytical). These three waterbird taxa represent distinct functional groups and have unique guano nutrient contents (Table S1; Adhurya et al., 2020; Hahn et al., 2007; Petkuviene et al., 2019; Pettigrew et al., 1997). Nutrient content of guano used in our experiments is reported in Table 1. Mallards are migratory, omnivorous, dabbling ducks with high N:P guano. Geese are migratory and resident herbivores which have high N:P. Cormorants are piscivorous waterbirds that are present throughout most of the year and have low N:P guano, with high P content.

**TABLE 1:** Nutrient content of composite guano samples from three waterbird species used in our experiments (mean ± 1 standard deviation).

Guano type	Sample size (*n*)	Total *N* (mg g^−1^ dw)	Total P (mg g^−1^ dw)	Total C (mg g^−1^ dw)	N:P (molar)
Mallard	3	58.2 (±5.9)	8.7 (±2.3)	317 (±52.4)	14.8
Canada goose	1	24.9	4.41	365	12.5
Double‐crested cormorant	1	45.2	102.1	137	0.98

*Note*: Sample size indicates the number of composite samples.

### 2.3 Wetland sediment uptake of guano‐derived nutrients

To understand how rapidly wetland sediments can remove nutrients released from waterbird guano contributions to the water column (via microbial and geochemical processes), we added fresh waterbird guano to sediment‐surface water microcosms (Figure S2). We gathered sediment and surface water from the wetland complex at OWC (41.378, −82.512) in early October 2022. We completed this experiment in autumn to match the time of year when waterbirds are present at the highest densities. We constructed microcosms using 7.62 cm diameter polycarbonate core tubes that we filled with ~15 cm of sediment and 500 mL of filtered (Pall, AE 1 μm) surface water and incubated microcosms under dark conditions at 20°C. We let sediment in the microcosms settle for ~24 h, with half receiving an anoxic treatment (surface water was gently bubbled with N_2_ gas), and half receiving an oxic treatment (surface water was gently bubbled with air). This allowed us to capture how wetland sediments process guano‐derived nutrients under a range of normal wetland oxygen conditions. We added the following guano treatments to both oxic and anoxic microcosms (*n* = 3 per treatment): no‐addition control, mallard low (+0.5 g wet weight [ww]), mallard medium (+1 g ww), mallard high (+1.5 g ww), goose medium (+1 g ww), and cormorant medium (+0.11 g dry weight, ~1 g ww equivalent based on 0.1 dw:ww assumption from collected wet mallard guano, Figure S2). We selected these addition levels to increase background TN concentrations by 10–20×, which resulted in increases in background TP concentrations by 10–200×. Our treatment levels were constrained by logistical limitations of microcosms available and quantities of guano. To maximize cross‐taxa comparisons of sediment uptake of guano, we prioritized using a single guano addition mass for all three species. Because we had more mallard guano available, we included additional low and high treatments for mallards to assess whether nutrient uptake varied with guano quantity.

We collected surface water seven times over 11 days, with more frequent sampling during the first few days when we expected rapid changes to occur. Sample times were relative to when guano was added and occurred at 0 h (immediately prior to guano addition), 4 h, 1 day, 2 days, 4 days, 6 days, and 10 days. We collected, filtered (0.45 μm PES, Sterlitech), and froze 50 mL of water from each microcosm, and immediately replaced this sampled volume with filtered (Pall, AE 1 μm) site water. We analysed water samples for TP, TN, ammonium (NH_4_^+^‐N), nitrate (NO_3_^−^‐N), and soluble reactive phosphorus (SRP) concentrations on a flow‐injection nutrient analyser (Skalar San++). Dissolved inorganic nitrogen (DIN) was calculated by taking the sum of NH_4_^+^‐N and NO_3_^−^‐N.

### 2.4 Algae community response to guano additions

We investigated how guano‐derived nutrients can change phytoplankton community composition by adding three masses of the three guano types to clear bottles with OWC surface water and incubating these bottles under lighted conditions (Figure S2). Treatments included (*n* = 3 per treatment): no‐addition control, mallard low (+0.5 g ww), mallard medium (+1 g ww), mallard high (+1.5 g ww), goose low (+0.5 g ww), goose medium (+1 g ww), goose high (+1.5 g ww), cormorant low (+0.055 g dw, ~0.5 g ww equivalent), cormorant medium (+0.11 g dw, ~1 g ww equivalent), and cormorant high (+0.16 g dw, ~1.5 g ww equivalent). We inverted the bottles once daily and otherwise left them uncapped to allow for gas exchange. After approximately 4 days, we collected and preserved water samples with glutaraldehyde for algal community composition analysis. Algae samples were counted using an inverted phase contrast microscope (Zeiss) under 100× total magnification. At least 400 natural units were identified to the lowest practical taxonomic unit (typically genus, with all distinct morphospecies identified) per sample and all algal cells in each natural unit were counted. We also collected and filtered 50–150 mL of water from each sample through 1 μm glass fibre filters, and then froze and extracted chlorophyll *a*, as a metric of algal biomass, from the filters using an ethanol extraction. The guano:water ratios used in this experiment are likely higher than those experienced on an hourly basis given average waterbird densities and dropping rates in these wetlands, but could also be lower than the guano:water ratios experienced locally in certain conditions (i.e. high waterbird densities, shallow water depth, limited flow).

### 2.5 Estimating total waterbird‐nutrient fluxes in coastal wetlands

We conducted waterbird counts in each wetland approximately every other week from November 2022 through October 2023 starting at ~half an hour after sunrise (start times between 6:15–8:00 AM and completed by 9:00–11:00 AM) to get a full year of waterbird densities to inform annual nutrient load calculations for both OWC and Turtle Creek. We conducted manual point counts at predetermined locations from the shore and counts over each wetland using a drone to capture videos at 50–60 metres high (Autel Evo II Pro V2; Figure S3; Table S2) to non‐intrusively count and identify waterbirds in areas where wetland vegetation obscured views from shore (Dundas et al., 2021; McEvoy et al., 2016). In areas where both point and drone counts were taken, we took only the highest count for each species from either method so as not to double count individuals. The drone was better suited to capture species that occupied areas with dense wetland vegetation and point counts ensured that we captured waterbirds that vacated the study site at first arrival. Birds were identified to species, or, when necessary (<5% of counts), closest function group.

### 2.6 Data analysis

We completed all statistics in R (version 4.3.2) and created all graphs with *ggplot2* (R Core Team, 2023; Wickham, 2009). For the wetland sediment uptake experiment, we calculated exponential decay rates for each guano treatment to understand nutrient (i.e. TP, SRP, TN, and DIN) removal rates. We fit a linear‐mixed effects model (*lme4* package; Bates et al., 2015), with log nutrient concentration as the response variable and time, guano treatment, oxygen treatment, and their interactions as fixed effects. We included random intercepts of unique core ID to account for repeated measures through time. Because decay rates are the slope of concentration over time, we inferred treatment effects on decay rates using the interactions of guano and oxygen treatment with time, but for simplification, henceforth omit reference of time when describing results. Using the mean exponential decay rate constants, we calculated the number of days it took until 50% removal was reached. We also calculated the percentage of each nutrient removed by taking the lowest mass remaining (from the two last timepoints of the experiment) and dividing it by the largest mass present after guano addition (from Timepoints 1 and 2) to make scaled comparisons about how effectively wetland sediment can remove guano nutrient inputs from different species and at different addition levels. We used a linear model comparing the percent of each nutrient removed across guano treatment, oxygen treatment, addition level treatment, and the interaction of guano and oxygen treatments. For all models, we confirmed assumptions of normality of equal residuals using diagnostic plots of residuals. We assessed post‐hoc pairwise differences with the *emmeans* package in R (Lenth, 2023).

Next, we compared how waterbird guano shapes major algal divisions using an ANOVA comparing diatom, green algae, and cyanobacteria proportions across guano treatments. Proportion data were both log and arcsine transformed to normalize residuals. We also compared chlorophyll *a* (log‐transformed) between guano treatments using an ANOVA. We assessed post‐hoc differences between treatments using Tukey tests when main effects were significant (*p* < 0.05). To examine how different waterbirds shape algal communities, we used a redundancy analysis (RDA) comparing algal communities between guano addition treatments using the *vegan* package in R (Oksanen et al., 2022). We rarefied algal counts to standardize sampling effort across all samples and then Hellinger transformed the data (Borcard et al., 2011; Legendre & Gallagher, 2001). We then identified algal taxa with a goodness‐of‐fit of >40% with the first two RDA axes as having strong associations with the guano treatments.

### 2.7 Calculations of annual waterbird loading and recycling

Guano contributions represent a mix of recycled nutrients and subsidies, depending on waterbird feeding behaviour and movement. Some species forage outside of the wetland and deposit external nutrients into the wetland as subsidies, while other species feed and reside within the wetland and recycle local resources. Many species do both, making it difficult to distinguish between subsidized and recycled nutrients. For simplicity, we refer to all guano nutrient contributions as waterbird fluxes because our estimates do not separate external subsidies from internal recycling. Regardless of origin, guano‐derived nutrients enhance the bioavailability of nutrients by mineralizing organic nutrients into more usable, inorganic forms as they are digested and excreted.

To estimate waterbird‐nutrient fluxes in both wetlands, we first calculated the daily dropping rates (dry weight day^−1^; Scherer et al., 1995) as 2.25% of the average adult weight of each bird species or functional group (Sibley, 2016), with the exception of Canada goose, where we used 3.7% of their average body weight (following Kear, 1963). These estimated dropping rates are conservative estimates across species (Scherer et al., 1995). For unsampled days, we used density estimates gathered on the nearest Julian date. For each day, we multiplied dropping rates per bird by nutrient content of the guano gathered from our own measurements (mallard, Canada goose, double‐crested cormorant) or from other published sources (Adhurya et al., 2020; Hahn et al., 2007; Irick et al., 2015; Marion et al., 1994; Pettigrew et al., 1997) to get a daily waterbird flux per individual waterbird (see Table S2). For OWC, we scaled the calculated waterbird‐nutrient fluxes to the whole wetland area, where our waterbird counts covered a smaller area than the whole wetland extent (65% sampled). We report our waterbird‐nutrient fluxes as monthly and yearly sums with separations by waterbird groups (i.e. dabbling duck, diving duck, goose, piscivore, and wading) for each wetland. Our estimates assume that waterbird densities on any given sampled day were representative of the entire day and days in between counts, and that species use the wetland similarly throughout the day relative to when we surveyed. Therefore, these are coarse estimates of how much N and P is recycled and loaded into the wetlands. After estimating the total waterbird‐nutrient flux in OWC, we compared it to external loading from the OWC watershed, calculated from published concentration and flow data for the 2023 water year (11,546 kg of total P year^−1^ and 100,376 kg of total N year^−1^; McMurray et al., 2024). In Turtle Creek, water‐level control structures modify the connection of the stream (the water source) to the wetland. Managers only allowed flow into the wetland to occur once in the 2024 water year, resulting in a load of ~20 kg of TP and 313 kg of TN to the wetland, while the stream load (source for the wetland) was roughly 3175 kg of TP and 34,000 kg of TN (Lake Erie and Aquatic Research Network: Wetlands and Water Quality Group, 2025).

## 3 RESULTS

### 3.1 Wetland sediment uptake of guano‐derived nutrients

TP, SRP, and TN declined in sediment microcosms over time across all guano types in oxic treatments (Figure 2a–c), while DIN concentrations remained relatively stable after guano additions (Figure 2d). Decay rates for TP, SRP, TN, and DIN differed across guano treatments, with all guano treatments having different decay rates than in controls (all *p* < 0.001, Figure 2, Table S3). Mallard treatments generally had the highest decay rates across all nutrient forms (TP, SRP, TN, and DIN; Table 2). For TP, cormorant treatments had the second highest decay rate, followed by goose treatments, while this was inverse for SRP, TN, and DIN. Decay rates were higher under oxic conditions than anoxic treatments for TP (*p* < 0.001) and SRP (*p* = 0.002), but were similar under both oxygen conditions for TN (*p* = 0.10) and DIN (*p* = 0.76; Figure 2). The interaction between guano type and oxygen treatment was only significant for TP (*p* < 0.001), with controls having similar decay rates under oxic and anoxic treatments, while guano treatments were different across oxic and anoxic treatments.

**FIGURE 2:**
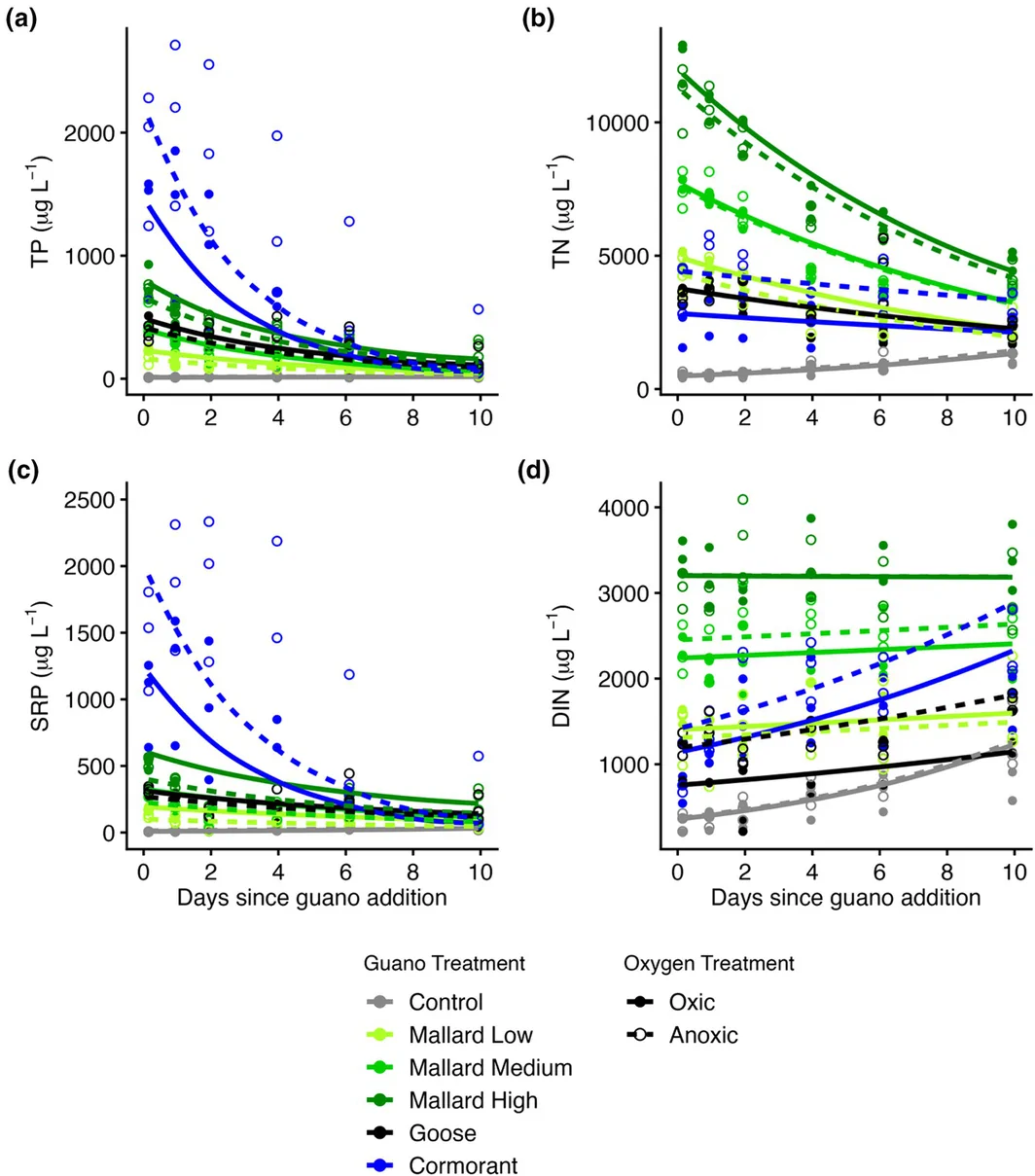
Concentrations of surface water (a) total phosphorus (TP), (b) total nitrogen (TN), (c) soluble reactive phosphorus (SRP), and (d) dissolved inorganic nitrogen (DIN) over time in wetland sediment‐surface water microcosms with oxic or anoxic overlying water following guano additions from three different waterbird species. Lines display predicted exponential decay rates for each guano and oxygen treatment. TP, TN, SRP, and DIN decay rates all differed across guano treatments, TP and SRP had higher decay rates in oxic treatments than anoxic treatments, and TP varied among guano × oxygen treatments (all *p* < 0.001). Decay rates also differed (all *p* < 0.001) between guano addition levels (mallard treatments only) for TP, TN, SRP, and DIN and among level × oxygen treatments for TP and DIN.

**TABLE 2:** Initial concentrations (C_0_) measured after guano treatment, the number of days until 50% removal is reached, and the exponential decay rate (k) for each guano addition treatment in wetland sediment‐surface water microcosms.

Treatment			C₀ (μg L^−1^)				Days until 50% removal				k (days^−1^)			
Guano	Addition	Oxygen	TP	SRP	TN	DIN	TP	SRP	TN	DIN	TP	SRP	TN	DIN
Control	‐‐	Oxic	11	8.8	480	360	−20.0	−6.5	−7.6	−6.6	−0.035	−0.110	−0.091	−0.110
Control	‐‐	Anoxic	13	13.0	520	370	−13.0	−4.7	−6.2	−5.0	−0.054	−0.150	−0.110	−0.140
Mallard	Low	Oxic	230	190.0	5000	1400	3.3	5.2	8.1	−73.0	0.210	0.130	0.086	−0.010
Mallard	Low	Anoxic	160	100.0	4300	1300	5.8	16.0	9.3	−42.0	0.120	0.044	0.074	−0.016
Mallard	Medium	Oxic	400	330.0	7800	2200	2.6	4.5	7.3	−160.0	0.260	0.150	0.094	−0.004
Mallard	Medium	Anoxic	410	230.0	7600	2500	4.9	10.0	8.6	−69.0	0.140	0.068	0.081	−0.010
Mallard	High	Oxic	800	610.0	12,000	3200	2.0	3.0	7.2	−130.0	0.350	0.230	0.096	−0.005
Mallard	High	Anoxic	670	410.0	11,000	3200	6.9	14.0	6.6	100.0	0.100	0.049	0.100	0.007
Goose	‐‐	Oxic	490	310.0	3800	750	2.5	4.1	9.9	−13.0	0.280	0.170	0.070	−0.055
Goose	‐‐	Anoxic	420	270.0	3800	1200	7.3	21.0	20.0	−26.0	0.094	0.033	0.035	−0.027
Cormorant	‐‐	Oxic	1500	1200.0	2800	1100	1.7	2.0	31.0	−10.0	0.410	0.350	0.023	−0.070
Cormorant	‐‐	Anoxic	2200	2000.0	4400	1400	2.5	2.7	20.0	−9.4	0.280	0.250	0.035	−0.074

*Note*: Decay rates for all guano treatments were different than the control of the same oxygen treatment (all *p* < 0.05).

The amount of guano added (mallard treatments only) significantly influenced decay rates for all nutrient forms (*p* < 0.001), while the interaction of addition level and oxygen treatment was only significant for TP (*p* < 0.001) and DIN (*p* = 0.03, Figure 2, Table S3). Addition levels displayed higher decay rates than controls (all *p* < 0.001) but decay rates were similar among low–high treatments. Although not significant, high addition levels generally induced the highest decay rates, and low addition levels generally induced the lowest decay rates. Post‐hoc comparisons of TP decay rates revealed that all levels of guano addition were similar under anoxic conditions (all *p* = 0.45), but generally differed under oxic conditions (*p* < 0.001). For DIN, decay rates were different under oxic and anoxic treatments in control treatments (*p* < 0.001), but similar under oxic and anoxic treatments across the remaining addition levels. By using the decay rates to calculate the amount of time until 50% of added guano nutrients were removed, we found that removal was typically faster under anoxic conditions than oxic conditions (Table 2). Generally, goose treatments had some of the longest calculated time until 50% removal across all nutrient forms (except DIN, which increased over time in all treatments). Mallard treatments had some of the shortest removal times, and cormorant treatments were intermediate and were the least variable (Table 2).

TP removal (i.e. percent removed across 10 days) did not differ between guano type (*p* = 0.085), addition level (mallard only, *p* = 0.43), or the oxygen‐guano treatment interaction (*p* = 0.33), but did differ between oxygen treatments (*p* < 0.001), with higher removal in oxic treatments than anoxic treatments (Figure 3a). Overall, most of the TP added was removed across all oxic treatments (average 92.9%), with less removed in anoxic treatments (average 74.4%). SRP removal differed between guano treatments (*p* = 0.004), oxygen treatment (*p* < 0.001), and the interaction of guano and oxygen treatments (*p* = 0.037, Figure 3c). A greater proportion of SRP was removed in the cormorant treatment than the geese treatment (*p* = 0.003) but SRP removal was similar between the cormorant and mallard treatments. SRP removal in both goose and mallard treatments was higher in oxic treatments than anoxic treatments (both *p* = 0.003), but was similar between oxygen treatments for the cormorant treatment (*p* = 0.995). Different guano addition levels of mallard treatments did not have different SRP removal (*p* = 0.75). TN removal differed by guano type (*p* < 0.001) but not by oxygen treatment (*p* = 0.23), addition level (mallards only, *p* = 0.23) or oxygen‐guano treatment interaction (*p* = 0.32, Figure 3b). TN removal was lower in cormorant treatments than both mallard and goose treatments. Because DIN increased over time, we could not assess differences in removal. We observed a ~25% increase in mallard treatments (expect the high treatment which had ~25% removal) and a 50%–90% increase in goose and cormorant treatments. Full model outputs are included in Table S4.

**FIGURE 3:**
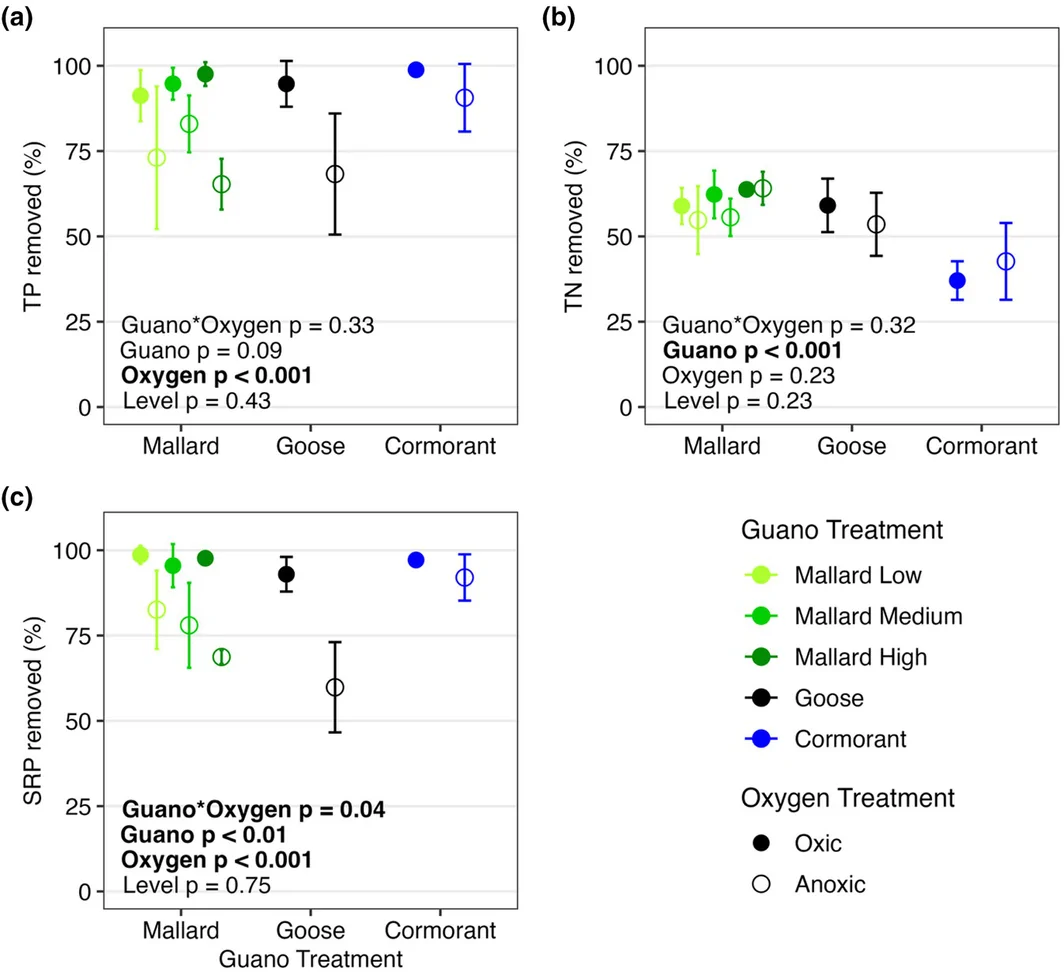
Percent of added guano (a) total phosphorus (TP), (b) total nitrogen (TN) and, (c) soluble reactive phosphorus (SRP) removed (± standard deviation) after 10 days in wetland sediment‐surface water mesocosms across guano, oxygen and level (mallard only) treatments. DIN increased over time, and therefore, removal could not be assessed. The *p*‐values for each factor from linear model analyses are reported.

### 3.2 Algae community response to guano additions

Algal growth differed across guano type (*p* < 0.001) and the interaction of guano and addition level (*p* = 0.014) but not addition level alone (*p* = 0.065, Figure 4b). Chlorophyll *a* concentrations were higher in all guano treatments than controls (*p* < 0.001) and higher in goose treatments than both mallard and cormorant treatments (*p* ≤ 0.001). The goose treatment was the only guano type that had greater chlorophyll *a* concentrations at higher guano additions, with the high addition resulting in higher chlorophyll *a* than the low addition (*p* = 0.017). Coarse‐scale taxonomic phytoplankton community composition differed slightly across guano treatments, with green algae and cyanobacteria proportions differing (*p* = 0.031 and *p* = 0.030, respectively, Figure 4a) but diatoms remaining similar across guano treatments (*p* = 0.065). Goose guano promoted higher proportions of green algae than control treatments (*p* = 0.019) and lower proportions of cyanobacteria than mallards and cormorants (*p* = 0.045 and *p* = 0.035, respectively). Coarse‐scale phytoplankton in cormorant and mallard treatments did not differ from controls.

**FIGURE 4:**
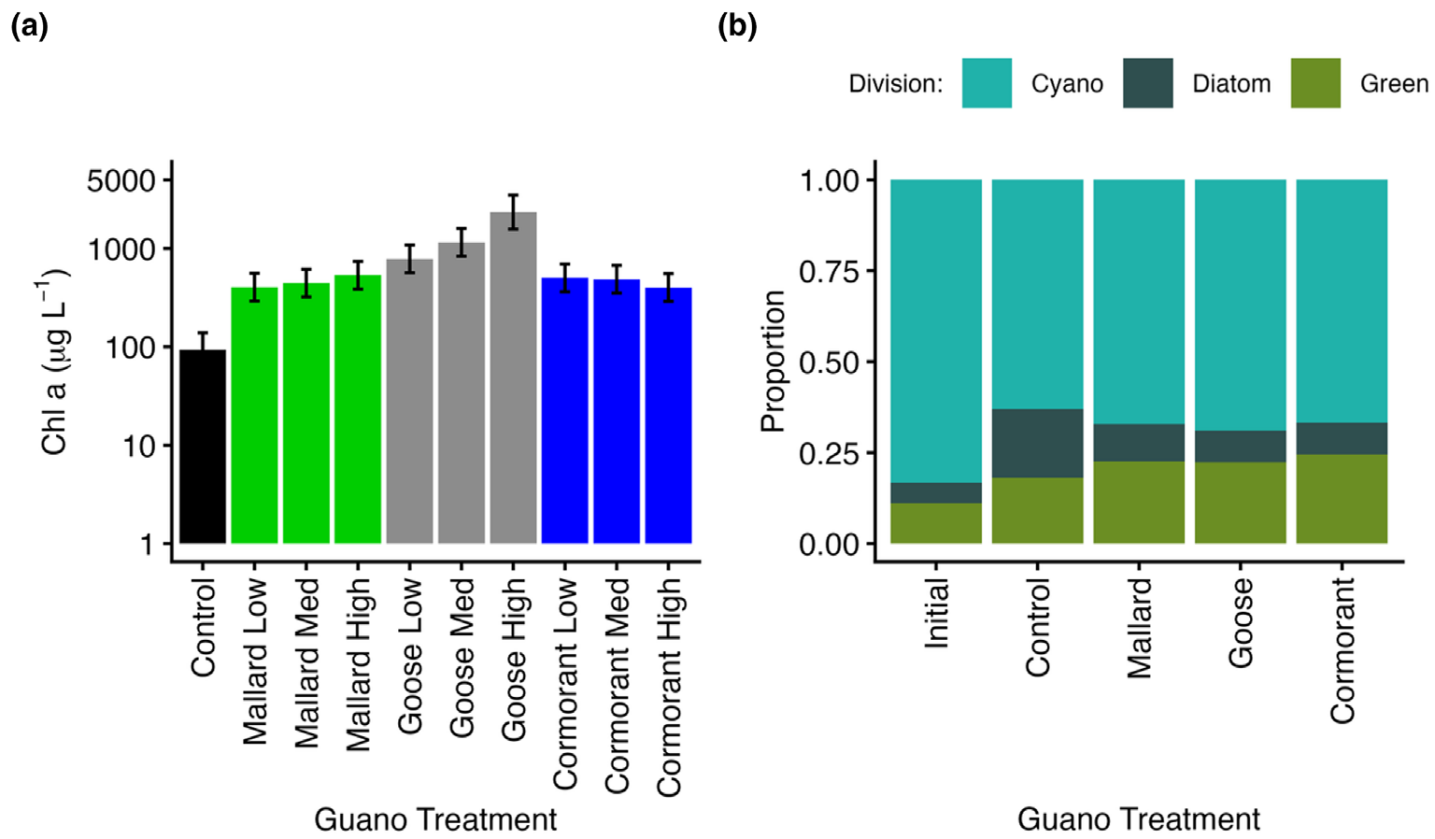
(a) Model estimated mean chlorophyll *a* concentrations (±95% confidence intervals, log‐transformed, *n* = 3) across guano and addition level treatments and (b) mean algal division proportions across guano treatments (*n* = 3 for initial and control, *n* = 6 for mallard, goose, and cormorant treatments).

We found 86 unique taxa across all treatments, and several cyanobacteria genera were the most numerous by cell count (e.g. *Aphanocapsa*, *Microcystis,* and *Merismopedia*). The RDA revealed that algal communities differed by guano treatment (*p* = 0.001, adjusted *r* ^2^ = 10.2%, *n* = 999 permutations, Figure 5). In the RDA model, 23.7% of the total variance in our algal communities was explained by constrained variables and 76.3% was explained by unconstrained variables. Control treatments are separated from all guano treatments along RDA1 axis, and differences among waterbird guano types are distinct along the RDA2 axis. The green algae genus *Pseudococcomyxa* was most associated with mallard treatments and the diatom *Synedra* (morphospecies 1) was most associated with cormorant treatments. No taxa were very strongly associated in the direction of the goose treatments, and the remaining taxa significantly explained by the constrained RDA axes (three diatom genera and the green algae genera *Crucigenia*) were more associated in the direction of the control treatments. Plots showing algal communities across RDA axes 1–3 are shown in Figure S4.

**FIGURE 5:**
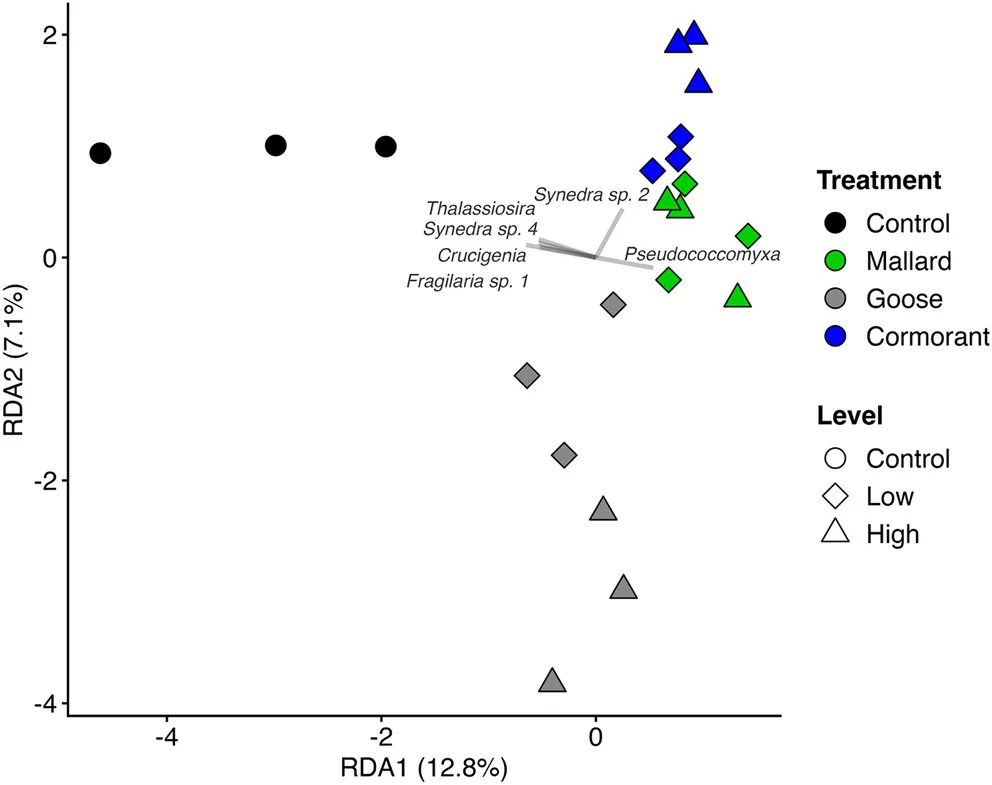
Redundancy analysis (RDA) biplot of algal community composition across guano treatments (*p* = 0.001, adj *r* ^2^ = 10.2%, *n* = 999 permutations). Listed taxa have a goodness‐of‐fit of greater than 40% with the RDA1 and RDA2 axes. Diatom taxa were *Synedra* sp. 2, *Synedra* sp. 4, *Fragilaria* sp. 1, and *Thalassiosira*. Green algae genera were *Pseudococcomyxa* and *Crucigenia*.

### 3.3 Annual waterbird‐nutrient flux estimates

Densities of waterbirds were similar in magnitude between the two study wetlands throughout most of the year, except during a migratory peak in October where densities were higher at Turtle Creek (Figure S5). The highest densities of dabbling ducks occurred in early autumn (October–November), diving ducks occurred in March, and piscivores (cormorants and white pelicans) occurred in late summer and early autumn (August–October). More migratory species were observed at Turtle Creek, resulting in higher densities in early autumn than at OWC. At most times of year, OWC had higher densities of wading birds (i.e. herons and egrets) than Turtle Creek, but the two wetlands had similar, high goose densities.

Patterns in seasonal waterbird use directly influenced waterbird‐nutrient fluxes, and overall, waterbird fluxes were greater in Turtle Creek than OWC (Table 3). In OWC, geese contributed the most TN and TP. Geese contributed ~2× as much TP and ~7.5× as much TN as any other waterbird group and made up ~50% of the total waterbird P flux and ~75% of the total waterbird N flux (Table 3). Piscivores (cormorants) and wading birds also contributed relevant amounts of the total waterbird fluxes of P (<30%) and N (<10%) at OWC. All other groups (dabbling and diving ducks) had lower magnitude fluxes (<5%). In Turtle Creek, piscivores contributed the highest TP flux (42% of the total waterbird flux) followed closely by geese (33%). Dabbling duck, diving duck, and wading bird fluxes collectively made up 25% of the total waterbird P flux. Similar to OWC, geese contributed the highest TN flux of any group in Turtle Creek at 50%, while dabbling ducks contributed 30%, and piscivores contributed 15%, and all other groups provided <5% of the total waterbird TN flux. In total, guano TP and TN fluxes were similar in magnitude at each wetland (molar N:P ~2.6 at each site; Table 3).

**TABLE 3:** Estimated annual total nitrogen (TN) and total phosphorus (TP) flux estimates for different waterbird groups at Old Woman Creek and Turtle Creek wetlands.

Site	Group	TP fluxes (kg year^−1^)	TN fluxes (kg year^−1^)
Old Woman Creek	Dabbling duck	2.45	8.07
	Diving duck	1.35	0.54
	Goose	71.9	131.6
	Piscivores	31.6	14.0
	Wading	42.7	18.3
	TOTAL	150	173
Turtle Creek	Dabbling duck	32.2	101.0
	Diving duck	15.4	6.22
	Goose	89.7	166.6
	Piscivores	114.5	50.7
	Wading	21.8	10.7
	TOTAL	274	335

Waterbird‐nutrient fluxes varied monthly, with the highest fluxes occurring when migrating dabbling ducks, piscivores and geese were more prevalent during the months of August–October in OWC and in October in Turtle Creek (Figure 6, Table S5). Nutrient fluxes were also high when there were high goose densities at both OWC (December–January) and at Turtle Creek Marsh (December, March, and April). While Turtle Creek had much higher fluxes during the peak of fall migration (October), fluxes across all other months were similar across both wetlands.

**FIGURE 6:**
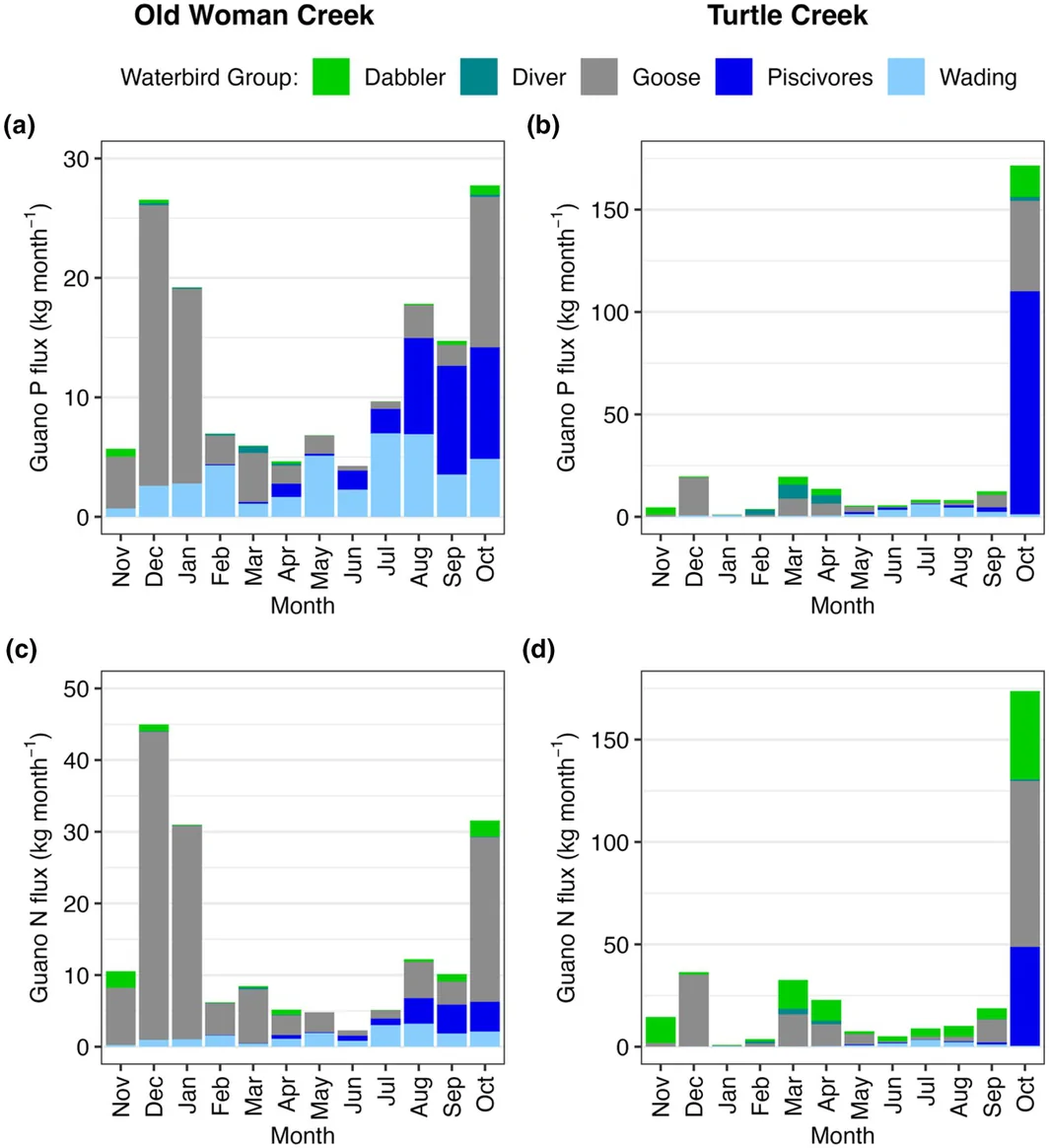
Estimated annual waterbird‐nutrient flux for different waterbird groups from November 2022 to October 2023. Waterbird fluxes of nitrogen (N) and phosphorus (P) at OWC (panels a and c) and Turtle Creek (panels b and d) are shown. Note the change in y‐axis scale between panels.

When compared to external loading from the OWC watershed in 2023 (11,546 kg of Total P year^−1^ and 100,376 kg of Total N year^−1^; McMurray et al., 2024), waterbird fluxes in OWC only amount to ~1.3% of the external TP load and ~0.2% of the external TN load. When compared to external loading to Turtle Creek in 2024 when water control structures were opened to bring in water once during the year (~20 kg of TP and 313 kg of TN), waterbirds provide 14× the external TP load and roughly equivalent TN as the external load; however, waterbirds only make up ~8.6% of the TP load and ~1% of the TN load from the stream that feeds the wetland (~3175 kg TP and 34,000 kg TN; Lake Erie and Aquatic Research Network: Wetlands and Water Quality Group, 2025).

## 4 DISCUSSION

Waterbird guano additions initially elevated nutrient concentrations in wetland sediment‐surface water microcosms. However, sediments rapidly removed most total P, and approximately half of the total N from the water column. This suggests that both waterbird guano‐derived fluxes and sediment biogeochemical processes collectively shape the net nutrient bioavailability in wetlands. Guano from different waterbird functional groups favoured distinct algal communities, largely driven by differences in total N content. Waterbird‐derived nutrient fluxes were seasonally variable (i.e. highest during fall migration), and may be locally important in providing bioavailable nutrients that support primary producers and food webs during nutrient‐limiting times (i.e. late summer and autumn) in wetlands (Cooper et al., 2016; Morrice et al., 2004). However, annual waterbird‐nutrient fluxes were negligible (<1.3%) compared with external loads from surrounding agriculture‐dominated watersheds, suggesting a minor role of waterbirds in nutrient recycling in these wetlands.

### 4.1 Wetland retention of guano‐derived nutrients

Wetlands remove nutrients contributed by waterbird guano over time, but the rate and amount at which they are removed depends on the nutrient (i.e. N or P). P additions were removed quickly and almost entirely within ~7 days, whereas N was not as effectively removed. We observed higher removal and retention of guano‐derived P (50%–60%) than N (25%–40%), which is consistent with other measured P and N fluxes in OWC (Richardson, 2018). OWC has high Fe:P ratios and high phosphorus sorption potential (Richardson, 2018), which suggests that the high P retention observed is likely driven by sorption to iron oxides. The low removal of N from added guano that we observed, paired with OWC having a water residence time of ~7.3 days (Herdendorf et al., 2006), likely results in substantial guano‐derived N being transported out of the wetland. Additionally, we observed increases in NO_3_^−^ concentrations over time in oxic conditions with limited declines in NH_4_^+^ concentrations. However, these temporal changes in DIN occurred at similar rates in the sediment controls (no guano addition), which may be a result of NH_4_^+^ porewater‐surface water exchange and subsequent nitrification to NO_3_^−^ occurring in the surface water. Increases in DIN could also be partially due to organic N and more recalcitrant fractions of guano slowly undergoing mineralization or degradation before being nitrified, but this is likely a small component on the time scale of this experiment. However, slow remineralization of nutrients in guano may be important on longer time scales because uric acid is a main component of bird guano (Szpak et al., 2012) and aerobic conditions are required for its degradation (Rouf & Lomprey, 1968), suggesting that N components of guano need oxic conditions and time before being fully remineralized. Despite high denitrification rates in OWC (McCarthy et al., 2007), we did not see high removal of N in any treatment. Furthermore, fewer nutrients were removed in anoxic treatments compared with the oxic treatments, which exemplifies the importance of water oxygen conditions in shaping nutrient removal processes. While most of the P from waterbird guano inputs will be retained by wetland sediments across the region, much of the N will likely be exported out of wetlands, into Lake Erie. Therefore, both waterbird guano‐derived nutrient fluxes and ecosystem nutrient removal rates collectively shape the net bioavailability of nutrients in these wetlands.

### 4.2 Waterbird species shape algal communities

We found that guano from different waterbird species distinctly shaped the biomass and composition of algal communities, consistent with previous studies (Petkuviene et al., 2019; Vizzini et al., 2016). All guano addition treatments facilitated higher phytoplankton biomass than control treatments; however, phytoplankton biomass only marginally increased with increasing amounts of mallard guano. This lack of variation among guano addition levels may indicate that nutrient conditions are saturated and growth rates are near maximums; other limiting factors exist, like light or micronutrient availability, or nutrients from guano are present mostly in recalcitrant forms (Larson et al., 2024). Interestingly, goose guano had 2–4× higher algal biomass at every guano addition level than any of the mallard and cormorant treatments, potentially due to the physical structure of goose droppings providing substrate for high biomass of filamentous green algae.

We found further support that nutrient additions from consumers (i.e. via waterbird guano) can shape phytoplankton communities (Kato et al., 2007). All guano additions caused relative increases in green algae with corresponding declines in relative abundance of both diatom and cyanobacteria compared with controls. Most of the algal taxa associated with guano treatments in the RDA were diatom genera, a unicellular green algae genus (*Pseudococcomyxa*) and a colonial green algae genus (*Crucigenia*). Some toxin‐producing cyanobacteria species like *Microcystis, Cylindrospermopsis,* and *Lyngba* were present in controls and treatments, but were at low abundances and not associated with any guano type. Overall, waterbird guano nutrient additions shaped distinct, diverse algal communities but did not cause increases in harmful cyanobacteria taxa. Mallard and cormorant guano promoted similar communities at both the division level and in the community RDA, despite having very different N:P. This is contrary to what we expected based on how N:P ratios in water typically shape phytoplankton communities (Reinl et al., 2022; Smith, 1983). Based on the RDA and the nutrient content of our three waterbird guano types, we suggest that guano N content may be driving algal community differences. Despite having different N:P, mallard and cormorant guano were similar in N content and distinct from goose guano N, while there were large differences in guano P content between all three waterbird species. Guano can also contain micronutrients like iron, manganese, cobalt, zinc, and nickel that are also important for algal growth and nutrient acquisition, and can shape algal communities (Larson et al., 2024; Stoll et al., 2024). Because sediment processes may result in incomplete removal of guano N inputs and N is driving community differences, waterbird fluxes may be locally important in changing phytoplankton communities and impact the food web (Vizzini et al., 2016).

### 4.3 Contributions of nutrients by waterbirds

Waterbird guano provides bioavailable nutrients to freshwater ecosystems, which can be used by primary producers and have bottom‐up effects on food webs. Estimated waterbird fluxes of N and P were similar in magnitude at the wetland scale, with a very low N:P of ~2.5. However, nutrients that are not removed or retained by wetland sediments and leave the wetland may have higher N:P because P is more efficiently removed from surface water than N. However, N‐limited algae could additionally remove N when biomass is high and influence the nutrient load N:P. Waterbird‐nutrient fluxes varied by waterbird group and through time, reflecting times when migrating waterfowl move through compared with when only residents remain. Annually, geese were the most numerous in OWC and contributed the highest N and P fluxes compared with other groups; however, piscivores and wading birds still contributed relevant P and N fluxes at OWC. Turtle Creek had high densities of dabbling ducks, but geese were still twice as high in density and contributed higher N and P fluxes comparatively because of their larger body size and higher excretion mass, despite having lower N and P content in their guano. At both wetlands, waterbirds generally contributed nutrients at similar proportions as we would expect based on their densities alone. An exception to this was wading birds which contributed ~10%–30% of the waterbird total P despite making up only 2%–7% of the waterbird community, highlighting the importance of guano nutrient content and stoichiometry in determining nutrient fluxes. Geese typically contributed more nutrients than cormorants due to higher densities, which suggests that concerns of high cormorant guano inputs in the Great Lakes region may be restricted to forested islands where the birds roost (Hebert et al., 2014) rather than all aquatic ecosystems that provide habitat. Goose management may be important for controlling nutrient inputs over time, especially if goose numbers continue to grow (Shirkey et al., 2018).

Fluxes were similar in magnitude between OWC and Turtle Creek across all months, except during October, which is the peak migratory period and when Turtle Creek experienced high migratory stopovers. This emphasizes how changing numbers of geese, piscivores and dabbling ducks throughout the year are important in shaping waterbird‐nutrient fluxes through time. The timing of maximum waterbird‐nutrient loads (i.e. fall and winter) are temporally distinct from when algal blooms form, which may leave time for sediment biogeochemical processes to retain and remove these added nutrients. However, microbial activity, and therefore nutrient removal, is also lower during colder months (Pomeroy & Deibel, 1986; Wilhelm et al., 2014), meaning that nutrients may be transported downstream before being microbially processed. Additionally, these wetlands are often ice‐covered in winter months, which may cause delayed pulses in nutrient inputs from waterbirds (especially geese) as the guano deposited on the ice enters the water during spring thaw.

The magnitude of nutrients contributed by waterbirds is small compared with external loads from landscape runoff. Total waterbird‐nutrient fluxes were <1.3% of the external loads, and thus in agriculture‐dominated watersheds, waterbirds may play only a small role in nutrient recycling and loading (Marion et al., 1994; Scherer et al., 1995). However, the importance of waterbird‐nutrient contributions may be higher in managed wetlands where water exchange is limited, like we observed in Turtle Creek. Overall, our findings suggest that wetland sediments can retain most of the P that is added by guano, but little of the N, and therefore, guano‐derived P is likely an even smaller component of the total nutrient load. It is also important to consider that guano inputs primarily supply PO_4_^3−^ and NH_4_^+^, which are highly bioavailable and can be rapidly used by primary producers, whereas external loads primarily consist of less bioavailable sediment‐bound P and NO_3_^−^. Thus, guano‐derived nutrient fluxes may be important locally to nutrient‐limited phytoplankton communities, but likely have little effect on total loading in agriculture‐dominated watersheds with continuous connection to the watershed. This suggests that the magnitude of waterbird‐nutrient effects is dependent on geographic region, wetland surface area coverage across the landscape, wetland habitat structure and management, and other nutrient inputs (Subalusky & Post, 2019).

### 4.4 Recycling and loading of guano to wetlands

Determining the sources of nutrients during waterbird foraging, and whether nutrients are transported within or across ecosystem boundaries, establishes guano as subsidies or recycled (Atkinson et al., 2017; Vanni, 2002). Most of the nutrients contributed by waterbirds in our study wetlands came from geese and cormorants, both of which primarily feed outside of the wetland, either in nearby agriculture fields (geese) or Lake Erie (cormorants) (Kitchell et al., 1999). Therefore, most of the guano‐derived nutrients we measured from these species were likely subsidies to wetlands (up to 65%–84%), with some nutrient recycling within and export out of the wetland occurring. Dabbling ducks, diving ducks, and wading birds often contributed intermediated amounts of nutrients via guano but primarily feed in wetlands with less movement between different wetlands (Kitchell et al., 1999). Therefore, nutrient fluxes from these groups (~15%–35%) are mostly recycled and remineralized into bioavailable forms that can influence primary producers and food webs and alter sediment‐surface water nutrient dynamics. Our findings expand upon the work examining the effects of consumer‐driven nutrient dynamics on ecosystem function in wetland ecosystems (Atkinson et al., 2017; Kitchell et al., 1999; Post et al., 1998; Subalusky & Post, 2019). The waterbird flux estimates we report provide a first look at the role of waterbirds on nutrient dynamics in Great Lakes wetlands and further work should ascertain what portion of nutrients are recycled or are subsidies to better inform whole‐ecosystem nutrient budgets for management purposes. Regardless of the path in which guano‐derived nutrients are released in wetlands, waterbirds provision bioavailable inorganic nutrients, which influence ecosystem function and support food webs.

### 4.5 Implications for management

Wetland cover across the landscape and habitat management strategies are important in determining waterbird densities, and influence the importance of waterbird‐nutrient fluxes compared with external loads from the watershed (Subalusky & Post, 2019). Studies have suggested that an increase in the number of wetland restoration projects within flyways will help geographically disperse waterfowl and other waterbirds and reduce high densities within individual wetlands (Post et al., 1998). Furthermore, focusing management strategies in diked wetlands that both maintain desired water levels and allow for more frequent water exchange will allow wetlands to be effectively managed for both waterbird habitat and nutrient removal (Andersen et al., 2003; Hansson et al., 2005). Understanding how nutrient fluxes from waterbirds play a role in nutrient budgets will help inform decisions for wetland restoration and management (i.e. water level and vegetation management) that prioritize both nutrient removal and waterbird habitat (Euliss et al., 2008). This paired management strategy is essential for not only reducing nutrients to downstream waters, but also conserving and restoring additional habitat for migratory waterfowl and waterbirds which are facing declines globally (U.S. Fish and Wildlife Service, 2022; Zimpfer et al., 2012).

## 5 CONCLUSIONS

Waterfowl and other waterbirds contribute nutrients to Great Lakes coastal wetlands through consumer‐driven nutrient recycling and subsidies, but most guano P additions can be retained by wetland sediments. Zoogeochemical impacts of waterbirds (i.e. guano inputs) can shape distinct algal communities, but this is likely driven more by guano N content than N:P ratios, with high N content guano (mallard and cormorant) promoting certain diatom species while low N goose guano promotes more green algae species. Waterbird flux contributions are negligible compared with external loads from the watershed, but may be locally and temporally important in stimulating the food web and shaping the algal community, especially in managed wetlands when flow connection is limited. Understanding the magnitude of nutrient contributions from waterbirds to wetlands in agricultural landscapes will help inform paired management strategies that work to prioritize simultaneous waterfowl and waterbird habitat and nutrient removal.

## AUTHOR CONTRIBUTIONS

Taylor C. Michael and David M. Costello conceived the ideas and designed methodology; Taylor C. Michael, Paisley Kostick, and Matthew A. Wuensch collected the data. Taylor C. Michael and David M. Costello analysed the data with support from Matthew A. Wuensch. Taylor C. Michael led the writing of the manuscript. All authors contributed drafts of the manuscript and gave final approval for publication.

## CONFLICT OF INTEREST STATEMENT

The authors report no conflicts of interest.

## STATEMENT ON INCLUSION

Our study was completed in Ohio, USA, where all authors reside and are stakeholders in the region. This study took place with the cooperation of local stakeholders (reserve and wetland managers). Whenever possible, we cited research conducted both in the local region and worldwide to put our study in context for the local region and for it to be useful on a global scale.

## Supporting information


**Figure S1.** Location of Old Woman Creek National Estuarine Research Reserve (OWC) and Turtle Creek Marsh along the coast of Lake Erie, and Winous Point Marsh along Sandusky Bay (labelled and denoted by white arrows). OWC is a site managed for nutrient retention and Turtle Creek is a site managed for waterfowl habitat. Waterbird guano was collected at OWC and Winous Point. The inset map places this region (yellow box) in the broader context of Lake Erie.
**Figure S2**. (A) Depiction of the wetland‐guano nutrient uptake experiment treatments. The mass of guano (wet weight) that was be added is shown above each microcosm. Each treatment was replicated three times. (B) Depiction of the bottle experiment assessing algae responses to guano addition treatments. The mass of guano that was be added is shown above each microcosm and depicted by white circles. Each treatment was replicated three times.
**Figure S3**. Old Woman Creek National Estuarine Research Reserve (left, 422 km^2^) and Turtle Creek Bay Marsh (right, 635 km^2^) with the waterbird drone survey area shaded in grey, point count locations indicated by stars, and wetland boundary lined in yellow.
**Figure S4**. Multiple redundancy analysis (RDA) axes plotted comparing algal community composition across guano treatments (*n* = 999 permutations).
**Figure S5**. Densities (per 100 km^2^) for the major waterbird groups at (A) Old Woman Creek National Estuarine Research Reserve (OWC) and (B) Turtle Creek Marsh at each sampling date. Stacked bars display the total density of waterbirds, divided by main groups of dabbling ducks, diving ducks, geese, piscivores (cormorants and white pelicans), and wading birds.
**Table S1**. Criteria used for calculating waterfowl nutrient fluxes to both Old Woman Creek National Estuarine Research Reserve (OWC) and Turtle Creek wetlands.
**Table S2**. Dates of waterfowl surveys and what count survey techniques (count, drone, or both) were used. No counts could be completed at Turtle Creek on 24 January 2023 due to construction occurring on the access roads.
**Table S3**. Statistical output from exponential decay linear‐mixed effects models.
**Table S4**. Statistical output from ANOVA models of per cent of nutrients removed.
**Table S5**. Estimated monthly waterbird total nitrogen (TN) and total phosphorus (TP) flux estimates for all waterbird groups from November 2022 through October 2023.

## Data Availability

Data are available from Zenodo repository https://doi.org/10.5281/zenodo.20495469 (Michael, 2026).
