# Supplementary material for: Waterbird zoogeochemical effects shape wetland nutrient dynamics and algal communities in Laurentian Great Lakes coastal freshwater wetlands
Source: J Anim Ecol. 2026 Jul 8;95(10):1887–902. doi: 10.1111/1365-2656.70308 (PMC13643184; doi:10.1111/1365-2656.70308)
Supplement: Supplementary file 1 [file JANE-95-1887-s001.docx]

**SUPPORTING INFORMATION**

Waterbird zoogeochemical effects shape wetland nutrient dynamics and algal communities in Laurentian Great Lakes coastal freshwater wetlands

Taylor C. Michael^1, 2*^, Paisley Kostick^1^, Matthew A. Wuensch^1,3^, and David M. Costello^1^

^1^Department of Biological Sciences, Kent State University, Kent, OH, USA 44242

^2^Department of Biological Sciences, University of Alabama, Tuscaloosa, AL, USA 35487

^3^Department of Biology, University of North Dakota, Grand Forks, ND, USA, 58202

^*^corresponding author (taylorcmichael4@gmail.com)

**Figures S1-S5**

**Tables S1-S5**

**Figure S1.** Location of Old Woman Creek National Estuarine Research Reserve (OWC) and Turtle Creek Marsh along the coast of Lake Erie, and Winous Point Marsh along Sandusky Bay (labeled and denoted by white arrows). OWC is a site managed for nutrient retention and Turtle Creek is a site managed for waterfowl habitat. Waterbird guano was collected at OWC and Winous Point. The inset map places this region (yellow box) in the broader context of Lake Erie.

**Table S1.** Criteria used for calculating waterfowl nutrient fluxes to both Old Woman Creek National Estuarine Research Reserve (OWC) and Turtle Creek wetlands.

| Species | Group | Scientific Name | Dropping dw as % Body Mass | Mean Body Mass (g) | Dropping Mass Per Bird  (g dw/d) | TN (mg/g dw) | TP (mg/g dw) | Guano Content Source |
| --- | --- | --- | --- | --- | --- | --- | --- | --- |
| Canada Goose | Goose | *Branta canadensis* | 3.7% | 4500 | 167 | 17.5 | 9.58 | Adhurya et al., 2020; Pettigrew et al., 1997, our measurements |
| Snow  Goose | Goose | *Anser caerulescens* | 2.3% | 2420 | 54.5 | 26.5 | 3.85 | Adhurya et al., 2020  average of Pink-footed goose and greater white-fronted goose |
| Trumpeter Swan | Goose | *Cygnus buccinator* | 2.3% | 10500 | 236 | 13.4 | 5.94 | Adhurya et al., 2020 |
| Mallard | Dabbler | *Anas platyrhynchos* | 2.3% | 1100 | 24.8 | 48.4 | 13.8 | Adhurya et al., 2020; Pettigrew et al., 1997, our measurements |
| American Black Duck | Dabbler | *Anas rubripes* | 2.3% | 1200 | 27.0 | 47.5 | 13.0 | our mallard measurements |
| Wood Duck | Dabbler | *Aix sponsa* | 2.3% | 600 | 13.5 | 47.5 | 13.0 | our mallard measurements |
| Gadwall | Dabbler | *Mareca strepera* | 2.3% | 910 | 20.5 | 47.5 | 13.0 | our mallard measurements |
| Northern Shoveler | Dabbler | *Spatula clypeata* | 2.3% | 610 | 13.7 | 47.5 | 13.0 | our mallard measurements |
| Blue Winged Teal | Dabbler | *Spatula discors* | 2.3% | 380 | 8.6 | 47.5 | 13.0 | our mallard measurements |
| Green Winged Teal | Dabbler | *Anas carolinensis* | 2.3% | 350 | 7.9 | 47.5 | 13.0 | our mallard measurements |
| Northern Pintail | Dabbler | *Anas acuta* | 2.3% | 800 | 18.0 | 47.5 | 13.0 | our mallard measurements |
| American Wigeon | Dabbler | *Mareca americana* | 2.3% | 720 | 16.2 | 47.5 | 13.0 | our mallard measurements |
| Dabbler sp. | Dabbler | *NA* | 2.3% | 741 | 16.7 | 47.5 | 13.0 | our mallard measurements |
| Hooded Merganser | Diver | *Lophodytes cucullatus* | 2.3% | 620 | 14.0 | 43.7 | 108 | average of carnivorous taxa from Hahn et al., 2007 and our measurements |
| Common Merganser | Diver | *Mergus merganser* | 2.3% | 1530 | 34.4 | 43.7 | 108 | average of carnivorous taxa from Hahn et al., 2007 and our measurements |
| Red Breasted Merganser | Diver | *Mergus serrator* | 2.3% | 1060 | 23.9 | 43.7 | 108 | average of carnivorous taxa from Hahn et al., 2007 and our measurements |
| Ring Necked Duck | Diver | *Aythya collaris* | 2.3% | 700 | 15.8 | 43.7 | 108 | average of carnivorous taxa from Hahn et al., 2007 and our measurements |
| Lesser Scaup | Diver | *Aythya affinis* | 2.3% | 830 | 18.7 | 43.7 | 108 | average of carnivorous taxa from Hahn et al., 2007 and our measurements |
| Bufflehead | Diver | *Bucephala albeola* | 2.3% | 380 | 8.6 | 43.7 | 108 | average of carnivorous taxa from Hahn et al., 2007 and our measurements |
| Ruddy Duck | Diver | *Oxyura jamaicensis* | 2.3% | 560 | 12.6 | 43.7 | 108 | average of carnivorous taxa from Hahn et al., 2007 and our measurements |
| Pied Billed Grebe | Diver | *Podilymbus podiceps* | 2.3% | 450 | 10.1 | 43.7 | 108 | average of carnivorous taxa from Hahn et al., 2007 and our measurements |
| American Coot | Dabbler/Diver | *Fulica americana* | 2.3% | 650 | 14.6 | 43.7 | 108 | average of carnivorous taxa from Hahn et al., 2007 and our measurements |
| Common Gallinule | Dabbler/Diver | *Gallinula galeata* | 2.3% | 312 | 7.0 | 43.7 | 108 | average of carnivorous taxa from Hahn et al., 2007 and our measurements |
| Great Blue Heron | Wading | *Ardea herodias* | 2.3% | 2900 | 65.3 | 42.1 | 115 | Hahn et al., 2007; Marion et al., 1994 |
| Great Egret | Wading | *Ardea alba* | 2.3% | 870 | 19.6 | 92 | 41.6 | Irick et al., 2015 |
| Black Crowned Night Heron | Wading | *Nycticorax nycticorax* | 2.3% | 870 | 19.6 | 92 | 41.6 | Irick et al., 2015 |
| Green Heron | Wading | *Butorides virescens* | 2.3% | 210 | 4.7 | 92 | 41.6 | Irick et al., 2015 |
| Double Crested Cormorant | Piscivore | *Phalacrocorax auritus* | 2.3% | 1700 | 38.3 | 45.2 | 102 | our measurements |
| White Pelican | Piscivore | *Pelecanus erythrorhynchos* | 2.3% | 7500 | 169 | 45.2 | 102 | our cormorant measurements |

**References:**

Adhurya, S., Das, S., & Ray, S. (2020). Guanotrophication by waterbirds in freshwater lakes: A review on ecosystem perspective. In P. K. Roy, X. Cao, X.-Z. Li, P. Das, & S. Deo (Eds.), *Mathematical Analysis and Applications in Modeling* (Vol. 302, pp. 253–269). Springer Singapore. https://doi.org/10.1007/978-981-15-0422-8_22

Hahn, S., Bauer, S., & Klaassen, M. (2007). Estimating the contribution of carnivorous waterbirds to nutrient loading in freshwater habitats. *Freshwater Biology*, *52*(12), 2421–2433. https://doi.org/10.1111/j.1365-2427.2007.01838.x

Irick, D. L., Gu, B., Li, Y. C., Inglett, P. W., Frederick, P. C., Ross, M. S., Wright, A. L., & Ewe, S. M. L. (2015). Wading bird guano enrichment of soil nutrients in tree islands of the Florida Everglades. *Science of The Total Environment*, *532*, 40–47. https://doi.org/10.1016/j.scitotenv.2015.05.097

Marion, L., Clergeau, P., Brient, L., & Bertru, G. (1994). The importance of avian-contributed nitrogen (N) and phosphorus (P) to Lake Grand-Lieu, France. *Hydrobiologia*, *279*(1), 133–147. https://doi.org/10.1007/BF00027848

Pettigrew, C. T., Hann, B. J., & Goldsborough, L. G. (1997). Waterfowl feces as a source of nutrients to a prairie wetland: Responses of microinvertebrates to experimental additions. *Hydrobiologia*, *362*, 55–66. <https://doi.org/10.1023/A:1003167219199>

**B**

**A**

**Figure S2.** (**A)** Depiction of the wetland-guano nutrient uptake experiment treatments. The mass of guano (wet weight) that was be added is shown above each microcosm. Each treatment was replicated three times. (**B)** Depiction of the bottle experiment assessing algae responses to guano addition treatments. The mass of guano that was be added is shown above each microcosm and depicted by white circles. Each treatment was replicated three times.

N

S

W

E

N

S

W

E

**Figure S3.** Old Woman Creek National Estuarine Research Reserve (left, 422 km^2^) and Turtle Creek Bay Marsh (right, 635 km^2^) with the waterbird drone survey area shaded in gray, point count locations indicated by stars, and wetland boundary lined in yellow.

**Table S2.** Dates of waterfowl surveys and what count survey techniques (count, drone, or both) were used. No counts could be completed at Turtle Creek on 24 January 2023 due to construction occurring on the access roads.

| Wetland Site | Date (MM/DD/YY) | Point Count | Drone Count |
| --- | --- | --- | --- |
| Old Woman Creek National Estuarine Research Reserve | 11/23/22 | Y | Y |
|  | 12/13/22 | Y | Y |
|  | 12/28/22 | Y | N |
|  | 1/24/23 | Y | N |
|  | 2/13/23 | Y | N |
|  | 3/05/23 | Y | N |
|  | 3/19/23 | Y | N |
|  | 4/11/23 | Y | N |
|  | 4/25/23 | Y | N |
|  | 5/08/23 | Y | N |
|  | 5/23/23 | Y | N |
|  | 6/21/23 | Y | N |
|  | 7/21/23 | Y | N |
|  | 8/16/23 | Y | N |
|  | 8/31/23 | Y | Y |
|  | 9/19/23 | Y | Y |
|  | 9/29/23 | Y | Y |
|  | 10/17/23 | Y | Y |
|  | 10/31/23 | Y | Y |
| Turtle Creek Wetland, Magee Marsh Wildlife Area | 11/23/22 | Y | Y |
|  | 12/13/22 | Y | Y |
|  | 12/28/22 | Y | N |
|  | 1/24/23 | N | N |
|  | 2/13/23 | Y | N |
|  | 3/05/23 | Y | N |
|  | 3/19/23 | Y | N |
|  | 4/11/23 | Y | N |
|  | 4/25/23 | Y | N |
|  | 5/08/23 | Y | N |
|  | 5/23/23 | Y | N |
|  | 6/21/23 | Y | N |
|  | 7/21/23 | Y | N |
|  | 8/16/23 | Y | N |
|  | 8/31/23 | Y | Y |
|  | 9/19/23 | Y | Y |
|  | 9/29/23 | Y | Y |
|  | 10/17/23 | Y | Y |
|  | 10/31/23 | Y | Y |

**Table S3.** Statistical output from exponential decay linear-mixed effects models.

| **Element** | **Term** | **Df 1** | **Df 2** | **F** | **p-value** |
| --- | --- | --- | --- | --- | --- |
| **Decay Rates** | | | | | |
| TP | GuanoTrt | 3 | 172 | 108.4 | <0.001 |
|  | OxygenTrt | 1 | 172 | 74.5 | <0.001 |
|  | GuanoTrt:OxygenTrt | 3 | 172 | 5.8 | <0.001 |
| SRP | GuanoTrt | 3 | 172 | 26.1 | <0.001 |
|  | OxygenTrt | 1 | 172 | 9.7 | 0.002 |
|  | GuanoTrt:OxygenTrt | 3 | 172 | 0.4 | 0.75 |
| TN | GuanoTrt | 3 | 172 | 145.5 | <0.001 |
|  | OxygenTrt | 1 | 172 | 2.7 | 0.10 |
|  | GuanoTrt:OxygenTrt | 3 | 172 | 1.7 | 0.17 |
| DIN | GuanoTrt | 3 | 172 | 42.7 | <0.001 |
|  | OxygenTrt | 1 | 172 | 0.1 | 0.76 |
|  | GuanoTrt:OxygenTrt | 3 | 172 | 1.8 | 0.15 |
| **Decay Rates: Addition Level** | | | | | |
| TP_level | LevelTrt | 3 | 112 | 84.2 | <0.001 |
|  | OxygenTrt | 1 | 112 | 79.7 | <0.001 |
|  | LevelTrt:OxygenTrt | 3 | 112 | 12.7 | <0.001 |
| SRP_level | LevelTrt | 3 | 112 | 11.3 | <0.001 |
|  | OxygenTrt | 1 | 112 | 7.4 | 0.007 |
|  | LevelTrt:OxygenTrt | 3 | 112 | 0.67 | 0.58 |
| TN_level | LevelTrt | 3 | 112 | 208.3 | <0.001 |
|  | OxygenTrt | 1 | 112 | 1.9 | 0.17 |
|  | LevelTrt:OxygenTrt | 3 | 112 | 0.9 | 0.44 |
| DIN_level | LevelTrt | 3 | 112 | 118.9 | <0.001 |
|  | OxygenTrt | 1 | 112 | 0.2 | 0.11 |
|  | LevelTrt:OxygenTrt | 3 | 112 | 3.1 | 0.03 |

**Table S4.** Statistical output from ANOVA models of percent of nutrients removed.

| **Element** | **Term** | **Df 1** | **Df 2** | **F** | **p-value** |
| --- | --- | --- | --- | --- | --- |
| TP | GuanoTrt | 2 | 22 | 2.755 | 0.085 |
|  | OxygenTrt | 1 | 22 | 24.279 | <0.001 |
|  | LevelTrt | 2 | 22 | 0.867 | 0.434 |
|  | GuanoTrt:OxygenTrt | 2 | 22 | 1.168 | 0.330 |
| SRP | GuanoTrt | 2 | 22 | 8.333 | 0.002 |
|  | OxygenTrt | 1 | 22 | 50.468 | <0.001 |
|  | LevelTrt | 2 | 22 | 1.372 | 0.274 |
|  | GuanoTrt:OxygenTrt | 2 | 22 | 4.914 | 0.0172 |
| TN | GuanoTrt | 2 | 22 | 18.041 | <0.001 |
|  | OxygenTrt | 1 | 22 | 0.652 | 0.428 |
|  | LevelTrt | 2 | 22 | 1.583 | 0.228 |
|  | GuanoTrt:OxygenTrt | 2 | 22 | 1.155 | 0.333 |

**Figure S4.** Multiple redundancy analysis (RDA) axes plotted comparing algal community composition across guano treatments (n=999 permutations).

**Figure S5.** Densities (per 100 km^2^) for the major waterbird groups at (A) Old Woman Creek National Estuarine Research Reserve (OWC) and (B) Turtle Creek Marsh at each sampling date. Stacked bars display the total density of waterbirds, divided by main groups of dabbling ducks, diving ducks, geese, piscivores (cormorants and white pelicans), and wading birds.

**Table S5.** Estimated monthly waterbird total nitrogen (TN) and total phosphorus (TP) flux estimates for all waterbird groups from November 2022 through October 2023.

| Site | Month | TP contributions (kg/ month) | TN contributions (kg/ month) |
| --- | --- | --- | --- |
| Old Woman  Creek Wetland | November | 5.7 | 10.5 |
|  | December | 26.5 | 45.0 |
|  | January | 19.2 | 31.0 |
|  | February | 7.0 | 6.2 |
|  | March | 6.0 | 8.5 |
|  | April | 4.6 | 5.2 |
|  | May | 6.8 | 4.8 |
|  | June | 4.3 | 2.3 |
|  | July | 9.7 | 5.1 |
|  | August | 17.8 | 12.2 |
|  | September | 14.7 | 10.1 |
|  | October | 27.7 | 31.6 |
| Turtle Creek  Marsh | November | 4.6 | 14.5 |
|  | December | 19.7 | 36.5 |
|  | January | 1.0 | 0.9 |
|  | February | 3.8 | 3.7 |
|  | March | 19.6 | 32.6 |
|  | April | 13.7 | 22.9 |
|  | May | 5.4 | 7.5 |
|  | June | 5.5 | 5.1 |
|  | July | 8.3 | 8.9 |
|  | August | 8.2 | 10.2 |
|  | September | 12.4 | 18.8 |
|  | October | 171.5 | 173.7 |
